# Transoral Laser-Assisted Total Laryngectomy: Expanding the TLM's World

**DOI:** 10.1155/2020/8827139

**Published:** 2020-09-26

**Authors:** Aslan Ahmadi, Saleh Mohebbi, Masoud Kazemi, Ayda Sanaei

**Affiliations:** ^1^ENT and Head & Neck Research Center and Department, The Five Sense Institute, Iran University of Medical Science, Tehran, Iran; ^2^ENT and Head & Neck Research Center and Department, Five Senses Health Research Institute, Hazrat Rasoul Akram Hospital, Tehran, Iran; ^3^ENT and Head & Neck Department, Shahid Beheshti University of Medical Science, Tehran, Iran

## Abstract

**Introduction:**

The introduction of laryngeal transoral procedures has created a shift in the treatment of laryngeal cancers towards the primary surgical management of patients. In this study, we aimed to evaluate the safety, efficacy, and feasibility of the transoral laser-assisted total laryngectomy (TLM-TL) in advanced laryngeal cancer. *Case presentation*. In this case report, we describe a case of a 50-year-old male patient presented to the otorhinolaryngology clinic with a history of hoarseness and odynophagia since 6 months. Based on the pathological and imaging findings, the diagnosis of stage IVa laryngeal squamous cell carcinoma with the involvement of the base, tongue, and left palatine tonsil was made for the patient, and transoral total laryngectomy with partial glossectomy via the TLM technique was planned.

**Result:**

The tumor was successfully resected by TLM-TL with clear surgical margins. No complication was observed after the surgery. Good functional recovery was obtained regarding swallowing and speech. The patient's oncologic and functional outcomes were evaluated for 2 years. Everything was satisfactory with good long-term cosmetic and laryngopharyngeal functional outcome and no sign of tumor recurrence.

**Conclusions:**

TLM-TL is a minimally invasive and cost-benefit endoscopic surgical procedure feasible in advanced laryngeal cancer with good long-term oncological and functional outcome. It could limit postoperative complications, mainly the incidence of pharyngocutaneous fistulae. It is also associated with better satisfaction after TL due to cosmetic benefits.

## 1. Introduction

An essential part of the management of laryngeal cancer is the preservation of function along with survival rate. These goals could be achieved via either surgical resection or chemoradiotherapy (CRT), and there is an ongoing debate on which approach is better in terms of oncological results and functional recovery [1, 2]. In the recent 20 years, the management of advanced larynx cancer underwent a significant evolution, and a gradual shift has been described from operative to nonoperative treatment [1, 3]. However, the CRT is associated with several significant side effects, including severe mucositis, pain, and dysphagia [4, 5]. Due to the significant toxicity of CRT regimens, there has been a resurgence of interest in the primary surgical management of patients with head and neck cancer [6]. Furthermore, several complications have been described for traditional total laryngectomy (TL) such as postoperative mortality and poor functional outcome [7].

Laryngeal transoral procedures have been developed to avoid the morbidity associated with either traditional open surgery approaches or CRT [8]. Several transoral techniques have been described for head and neck cancer, including transoral laser microsurgery (TLM), transoral robotic surgery (TORS), and transoral ultrasonic surgery (TOUSS) [1]. The major advantages of transoral procedure over the conventional open surgery include the comparable oncologic results to TL with good functional outcomes [9].

TLM is a preferred option for laryngeal cancers, and several studies have described this technique in intermediate and advanced tumors with low morbidity and reliable oncologic outcome [10]. In addition to the superiority of transoral technique to open TL, the transoral laser surgery in glottic cancer has several advantages over the radiotherapy treatments, mainly the potential for further laser surgery or radiotherapy in the event of local recurrence [11, 12]. TORS is a natural extension of the TLM technique [13].

Patients with extensive tumors (T3 and T4) usually require a TL procedure which could be performed via the transoral approach [14]. This study aimed to evaluate the safety, efficacy, and feasibility of TLM-TL.

## 2. Case Presentation

### 2.1. Clinical Presentation

**Figure 1:**
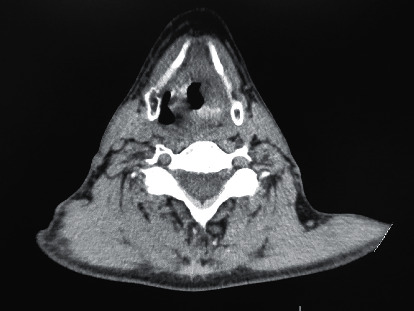
CT scan (axial view): an infiltrative mass at the left supraglottic area with extension to the left TVC.

A 50-year-old male patient presented to the otorhinolaryngology clinic, with a history of hoarseness and odynophagia since 6 months. He had a history of CRT with supraglottic SCC diagnosis a year ago (initial location of the tumor and both sides of the neck). He was a heavy smoker (60 packs per year) and had a positive history of opium use. On physical examination, no neck mass was observed. Laryngeal videostroboscopy and computed tomographic (CT) scan were performed demonstrating a huge laryngeal mass at the left posterior part of the tongue spreading to the glottis (Figure 1). The patient underwent direct laryngoscopy, and biopsy was taken from the base of the tongue. The histopathologic report revealed squamous cell carcinoma (SCC) of the larynx. According to pathological and imaging findings, the diagnosis of stage IVa laryngeal cancer (involvement of the base the tongue and right palatine tonsil) and transoral total laryngectomy with partial glossectomy via the TLM technique was planned for the patient.

### 2.2. Surgical Technique

The TLM was performed using a carbon dioxide (CO_2_) laser (SmartXide DOT®, DEKA Research & Development Corporation, Italy) with a 10600 nm laser beam spot. The CO_2_ laser was used as a “light scalpel” to excise a lesion with appropriate margins as an en bloc resection. To produce adequate cutting and hemostasis with limited thermal damage to surrounding tissues, a pulse delivery with high energy-short duration pulses was used. For vessel coagulation with a maximum diameter of 0.5 to 1, the laser was used. Bleeding from larger vessels was controlled by carefully performing electrocautery [15–17].

This technique is made of two minimally invasive procedures (cervical and transoral approaches).

After general anesthesia, the patient was placed in a supine position with neither shoulder elevation nor neck extension to achieve a wider surgical field. The initial incision was made 2 cm above the sternal notch. After the cervical fascia incision, the anterior jugular veins and infrahyoid muscles were transected. To expose and transect the isthmus of the thyroid, a suture was made between the infrahyoid and the musculocutaneous flap.

Following the isthmus transection (for releasing trachea and larynx), the thyroid lobes were released laterally from the trachea and the larynx. The procedure continued using an endoscope. A space was created under the sternohyoid muscles up to the hyoid bone level, referred to as the superior tunnel. While the superior tunnel was exposed with a Langenbeck retractor, the superior aspect of the sternohyoid muscle was transected on each side of the larynx. Then, the superior thyroid lobe was identified and released laterally from the larynx. The tunneling continued on its superior pathway upon sectioning the omohyoid muscle and the inferior insertion of the thyrohyoid muscle.

**Figure 2:**
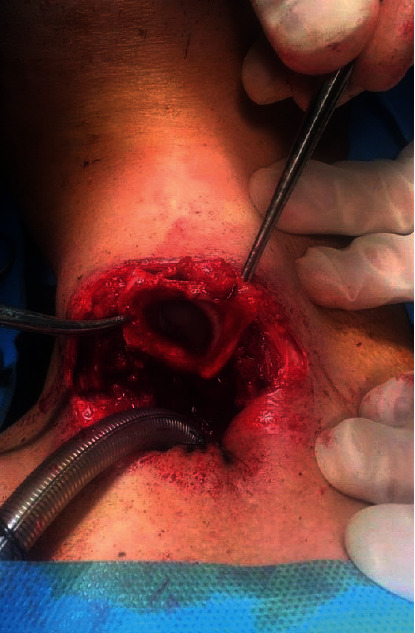
From the neck incision, the larynx is completely and circumferentially released.

For the next step, the trachea was opened, and the tracheal tube was placed. After fixing the trachea to the skin, an incision was made in the posterior tracheal wall at the superior ring level. This technique provides a beveled-cut tracheostomy leading to the better positioning of the voice prosthesis. The dissection continued through the space between the trachea and the esophagus, i.e., the inferior tunnel (Figure 2). The tunneling led to exposing the posterior cricoarytenoid muscles and the posterior aspect of the arytenoid cartilages. At this moment, the constrictor muscle was divided laterally at the level of the posterior border of the thyroid cartilage up to the superior cornu. Furthermore, the superior cornu was also released.

After stabilizing the stoma and dissection of the strap muscles down to the underlying laryngeal skeleton, transoral exposure of the larynx was started. At the first step, the access to larynx was secured using a Karl Storz 8588N Weerda Distending Laryngoscope. The limits of the mucosa resection were marked. Then, the vallecular mucosal incision was performed laterally towards the anterior incision and deeply towards the inferior tunnel using the CO_2_ laser. Furthermore, the pre-epiglottic space exposure was initiated with positioning the FK-blade behind the base of the tongue, and progression towards the inferior border of the hyoid bone was done. The superior tunnel was easily entered, and lateral dissection (cricoid mucosa and aryepiglottic fold) was performed towards the superior cornu with the CO_2_ laser, and superior laryngeal pedicles were ligated.

**Figure 3:**
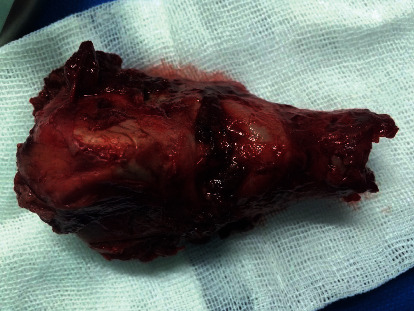
Larynx specimen after transoral laser-assisted total laryngectomy (TLM-TL).

Finally, with the traction of both superior cornu using two forceps, the larynx was completely removed, transorally (Figure 3). After the resection, the margins were evaluated via the frozen section analysis to ensure the complete oncological resection.

The pharyngeal mucosal closure was done using a 3/0 Vicryl with a modified gambee pattern. The distal part of the hypopharynx was sutured through a cervical incision. Then, the needle was passed through the cervical tunnel into the mouth, and the neopharynx was sutured from the distal to the proximal side. Finally, the suture ended in the base of the tongue. The proximal suture was performed using a long needle holder. The musculocutaneous flap was sutured to the anterior pharyngoesophageal wall to improve the contact between the layers, and a permanent tracheostomy was performed.

## 3. Result

The patient underwent TLM-TL, and the tumor was successfully resected with clear tumor margins. The total surgical time was 360 minutes, and one of the packed RBC was used during surgery. No ICU admission was required postoperatively. The hospital stay was short, and the patient was discharged 5 days after the surgery.

**Figure 4:**
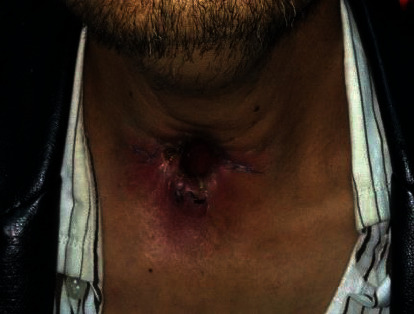
Three weeks after TLM-TL, compared to the conventional total laryngectomy, the aesthetic results are better, and there are no signs of pharyngocutaneous fistula.

No complication including pharyngocutaneous fistula (PCF), significant pain, and bleeding was observed after TLM-TL. There was no evidence of infection postoperatively and in the follow-up evaluations (Figure 4).

Good functional recovery was observed after surgery. The patient started oral feeding after five days. The nasogastric (NG) tube was preserved until a fully oral diet was possible. No sign of dysphagia was observed during the swallowing evaluation. Although a part of the base of the tongue was resected, oral motor exercises showed normal tongue movement.

The patient's oncologic and functional outcomes were evaluated in the regular phase for 2 years. Everything was satisfactory with good long-term cosmetic and laryngopharyngeal functional (speech with TEP voice and swallowing) outcome and no sign of tumor recurrence.

## 4. Discussion

The introduction of minimally invasive transoral techniques creates a paradigm shift in the treatment strategy for laryngeal and pharyngeal cancers. The TLM is the first transoral procedure which was developed by Steiner et al., and it is the most frequent organ preservation strategy since its development in 1988 [1].

Despite the major complication accompanying the TL surgery, it has a vital role in advanced laryngeal tumors. Traditional open TL techniques require extensive dissection and potentially associated with temporary or permanent morbidity. Since 2013, TORS-TL has been described by Lawson et al. as an alternative to traditional open TL with fewer morbidities and complications [18]. Since then, only a few studies have been conducted with further experience regarding the transoral TL procedure [3, 6, 19, 20]. Fernández-Fernández group described a new endoscopic, nonrobotic technique in 2016 to manage advanced supraglottic or pharyngeal tumors, using ultrasound as a scalpel. This technique could eliminate the need for a robotic platform to approach more advanced tumors [15].

In this case report, we used the same endoscopic approach as the Fernández-Fernández group with the only difference to be the use of the TLM technique instead of the TOUSS setup. This technique is made of two separate procedures, an endoscopic cervical approach through the tracheostomy and a transoral endoscopic approach to the larynx. We performed these two procedures without using expensive robotic systems. TLM-TL was associated with no complication and short hospital stay. We achieved a good oncological, functional, and cosmetic outcome which was preserved 2 years after the surgery.

The TLM-TL technique yielded several benefits for patients with advanced laryngeal cancer. With this technique, we could preserve the blood and neurologic supply to the pharynx and surrounding musculature. Other advantages of this method include better postoperation healing, minimum incision, no visible neck scar, and direct visualization of laryngeal mucosa and tumor distribution transorally under a microscopic view. Furthermore, the elimination of the robotic platform could lead to a lower cost of surgery as it was shown that using the TORS setup will increase the cost nearly twice compared to open TL [21].

One of the vital elements of TL surgery is the achievement of clear surgical margins. It has been described that a 50% decline in survival rate is expected if negative margins are not obtained. Thus, complete tumor resection could result in improved survival, which was observed in the present study. Additionally, shorter recovery time and limited bleeding were observed with TLM-TL compared to the open TL [3, 20, 22].

Open TL surgeries could be associated with significant neck dysesthesia, scarring, and fibrosis with a concomitant reduction in quality of life. Utilizing TLM-TL led to the prevention of huge neck scars and the avoidance of secondary repairing after TL. This results in a favorable cosmetic outcome, leading to a better quality of life. This trait is vital, especially after radiation therapy, which is associated with weaker skins. Furthermore, along with the growing population of female patients with head and neck cancers and the important role of aesthetics-related issues in this population, the cosmetic outcomes should be considered more than before [23–25].

Similar to TORS-TL and TOUSS-TL, TLM-TL is a feasible method in radiated patients as these patients could benefit from the minimal incisions. Furthermore, radiated patients could also benefit from TLM-TL due to lower PCF incidence. PCF is the most frequent complication after laryngectomy, and it has a significantly higher incidence in radiated patients. PCF is considered as an independent prognostic factor after TL which could cause longer hospital stay and an increase in treatment costs. Due to the abovementioned reasons, TLM-TL should be considered as a cost-benefit, safe procedure for salvage total laryngectomy without neck dissection in radiated patients [25–29].

It should be noted that due to the advanced stage of the patient's SCC cancer and the involvement of supraglottic areas, the surgical field was limited. Thus, to achieve the optimal outcome, TLM-TL is suggested to be performed on patients with tumors limited to the glottic region.

## 5. Conclusions

TML-TL is a minimally invasive and cost-benefit endoscopic surgical procedure feasible in advanced laryngeal cancer with good long-term oncological and functional outcome. It is also associated with a better quality of life after TL due to cosmetic benefits treatment.
